# Arthroscopic Treatment of a Posterior Labral Interposition after a Pediatric Hip Dislocation—A Case Report

**DOI:** 10.1055/s-0038-1661408

**Published:** 2018-07-13

**Authors:** Christiane Kruppa, Marcel Dudda, Thomas A. Schildhauer, Dominik Seybold

**Affiliations:** 1 Department of General and Trauma Surgery, BG University Hospital Bergmannsheil, Ruhr-University, Bochum, Germany; 2 Department of Orthopaedics and Traumatology, Universitatsklinikum Essen, Essen, Nordrhein-Westfalen, Germany

**Keywords:** hip dislocation, arthroscopy, loose body, labral tear

## Abstract

We report the case of a 13-year-old boy, who suffered a posterior hip dislocation from playing soccer. Closed reduction was performed urgently. Because of a nonconcentric hip after closed reduction, further imaging was done. An intra-articular bony fragment was identified. Arthroscopic treatment was performed. Through an anterior portal we were able to locate the intra-articular bony fragment, which was located within the region of the fovea. After lifting of the caudal enfolded labral complex, we were able to remove the fragment. Evidence of a grade 3 cartilage defect was present at the femoral head. We were able to reduce the enfolded posterior labral complex, which was stable afterwards without the necessity of additional suture fixations. The concentric hip reduction was confirmed on an anteroposterior view of the hip postoperatively. The patient was instructed to toe tip weight-bearing for 6 weeks with limited range of motion to 60° of hip flexion. Eight weeks after surgery, he was free of pain and discomforts. From our experience, the arthroscopic intervention after pediatric hip dislocation associated with intra-articular bony fragments or posterior labral complex injuries, represents to be a preferred minimally invasive method in contrast to open surgical procedures.

## Introduction

Consensus exists about emergency treatment of a pediatric hip dislocation. The hip may remain nonconcentric after reduction because of an intra-articular bony or cartilaginous loose fragment from the femoral head, an interposition of the labrum complex, or other soft tissue interpositions. 1 2 3 Different open reduction techniques have been described if concentric reduction fails, such as a posterior Kocher–Langenbeck approach to access the posterior joint space 1 or a surgical hip dislocation 4 5 to fully access the articular joint space. Due to an increased arthroscopic treatment of the adult hip, hip arthroscopy has been extended to include the pediatric population as well. 6 7 8 9 There have been a few reports focusing on arthroscopic treatment after a pediatric hip dislocation. 8 10 11

To add to the current literature, we will report a case of arthroscopic treatment of an intra-articular osteochondral fragment and labral tear in a 13-year-old boy.

## Patient and Technique

**Fig. 1:**
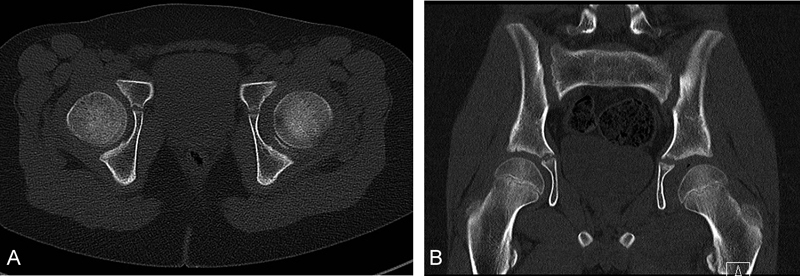
( **A** and **B** ) The computed tomography (CT) scans show the intra-articular bony fragment which caused the nonconcentric reduction of the left hip.

**Fig. 2:**
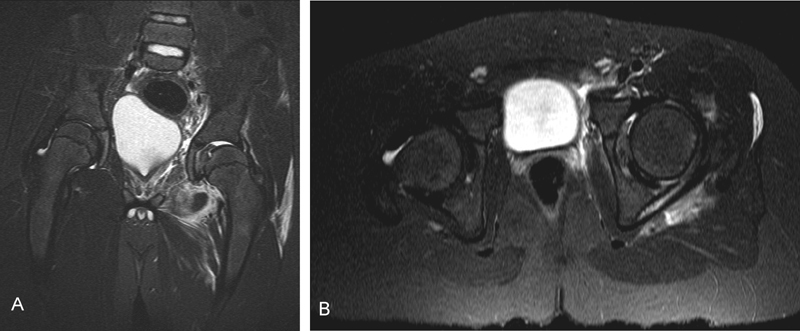
( **A** and **B** ) The magnetic resonance imaging (MRI) shows the nonconcentric hip with intra- and periarticular edema.

We report the case of a 13-year-old boy, who sustained a posterior left hip dislocation during a soccer duel. His weight was 47 kg and height was 152 cm. After an initial anteroposterior (AP) view, the hip was emergently reduced under sedation on the day of trauma in another surgical department by a pediatric surgeon. Because the postreduction AP view of his left hip showed a nonconcentric reduction, a computed tomography (CT) scan ( Figs. 1A and B ) and a magnetic resonance imaging (MRI) ( Figs. 2A and B ) of his left hip were performed. On CT and MRI scans, an intra-articular bony fragment, which was detached from the femoral head, was identified. Five days after the reduction, the patient was referred to our Department of General and Trauma Surgery for further treatment. The reasons for the delay of the referral are unknown. On physical examination, the patient exhibited sensory and motoric deficits to his sciatic nerve with two-fifths restricted strength of dorsal flexion of his left foot and hypaesthesia in the areas of the common peroneal and tibial nerves.

**Fig. 3:**
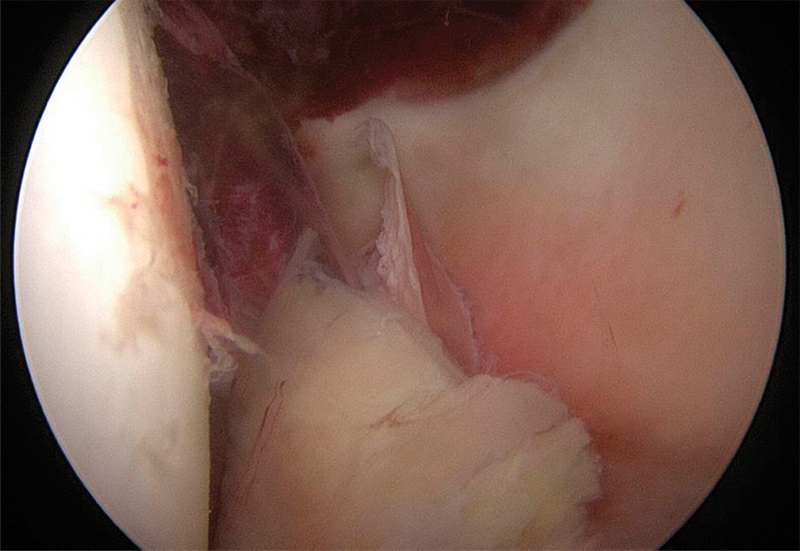
Small intra-articular osteochondral fragment, which was detached from the femoral head.

After careful evaluation of the images and consideration of the possible operative approaches, we decided to treat the patient arthroscopically. Operative treatment was performed on the sixth day postinjury. The patient was placed supine on a fracture table. Traction on the left leg and countertraction on the right leg was carefully applied until the joint space was adequately widened. Under fluoroscopic guidance, the hip was penetrated with a trocar needle for an anterolateral port and a 70° arthroscope was inserted over a guided wire. An additional anterior working port was established. The intra-articular hematoma was sectioned and debrided with a shaver. We were able to identify an osteochondral fragment in the fovea attached to the posterior labrum complex ( Fig. 3 ). A grade 3 chondral lesion based on the Outerbridge classification system (grade 0–4) 12 was seen on the femoral head as well as a small contusion of the posterior acetabular rim.

**Fig. 4:**
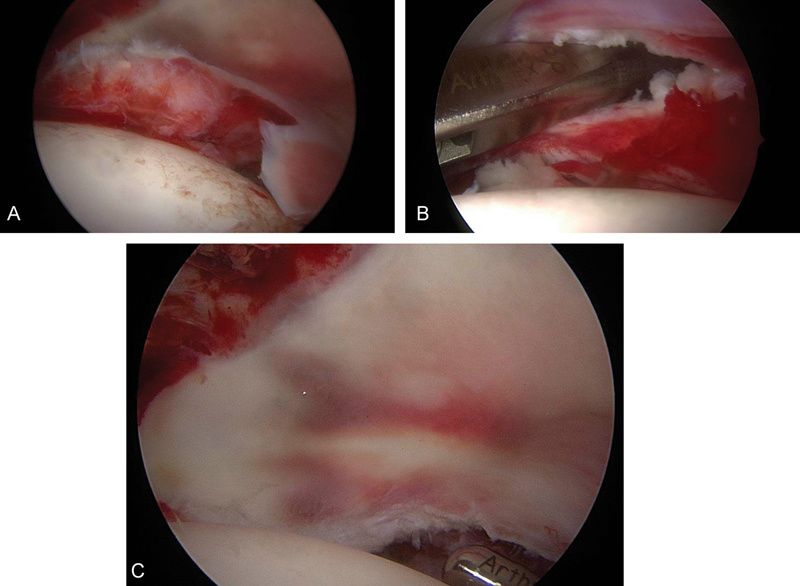
( **A** , **B** , and **C** ) Intraoperative images showing the enfolded labrum, which was reduced with careful manipulation with a hook. The labral complex was stable after reduction.

**Fig. 5:**
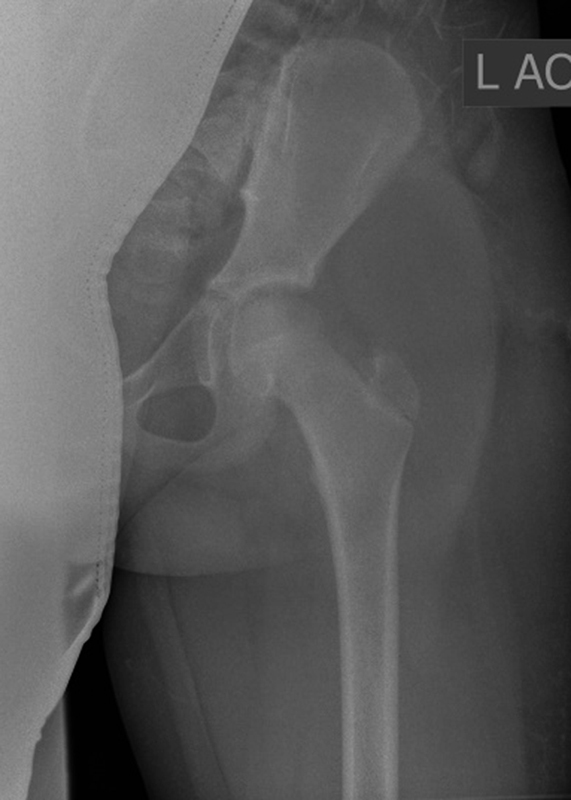
Anteroposterior (AP) radiograph shows the concentric reduction of the hip after arthroscopic treatment.

We additionally identified a tear at the posterior labral complex, which led to complete enfoldment of the posterior labrum. Both injuries were responsible for the nonconcentric reduction. With a biter the osteochondral fragment was removed, which during dislocation had obviously detached from the femoral head. Careful debridement of the labral tear was performed. Furthermore, we were able to manipulate the posterior labral complex with a hook until it was unfolded ( Figs. 4A–C ). Postreduction the labrum appeared to be stable without repeat enfoldments, and therefore no further suture anchors or wires were deemed necessary. Operative procedure time was 65 minutes. Postoperatively concentric hip reduction was confirmed with an AP radiograph of the left hip ( Fig. 5 ). The hip was clinically stable under full range of motion. The patient was instructed to toe tip weight-bearing for 6 weeks with restriction of hip flexion to a maximum of 60°. Eight weeks postsurgery, the patient was able to fully weight-bear without any pain or discomfort and had regained full range of hip motion. Remission of neurologic deficits was also noted.

## Discussion

Careful evaluation of concentric reduction is essential after closed reduction of a dislocated hip. Soft tissue interpositions, incarcerated labral tears, or intra-articular loose bodies can be frequently found postreduction. 11 Further imaging is required. Especially in the pediatric population, the MRI investigation is preferable to CT scan. In a series of 40 patients under 16 years old, Mayer et al reported that all surgically confirmed soft-tissue injuries were preoperatively identified on MRI, which also offered the advantage of absence of radiation in that age group. 13 CT scan may especially lead to underestimation of an osteochondral lesion at the posterior wall. 13 14 In the present case, a tear at the posterior labral complex, which lead to complete enfoldment of the posterior labrum, and an additional osteochondral fragment resulted in the nonconcentric joint after closed reduction of the hip. Similarly, treatment of an incarcerated acetabular labrum following reduction of a traumatic hip dislocation has been effectively reported with either refixation or excision of an osseo-labral fragment via hip arthroscopy in adults. 15

In the pediatric population, hip arthroscopy was initially used for various diseases, such as Perthes disease, slipped capital femoral epiphysis, or neuropathic subluxation. 16 Its use has been widened to include posttraumatic disorders. Morris et al reported seven patients (aged 8–17 years) who were arthroscopically treated after a traumatic hip dislocation. They described an entrapment of the posterior labrum with or without an attached bony fragment from the posterior acetabular wall as the predominant and consistent pathology in these patients. Removal of the fragment and reduction of the soft tissue without repair of the soft tissue or bony fragments was arthroscopically performed in all children. Concentric, stable reduction without the occurrence of an avascular necrosis, recurrent instability, or pain on follow-up was reported. 8 Intraoperative pathology reported in their series, was similar to the findings in our patient, except for the origin of the bony fragment, which belonged to the femoral head in our patient. Similar findings were reported by Wylie et al who performed hip arthroscopy after hip dislocation in 12 patients younger than 25 years (11–25 years). Eight patients were found to have intra-articular loose bodies, three patients with degenerative labral injuries, three full-thickness tears of the acetabular labrum, one separated labrum, and one incarcerated. In two patients, femoral articular cartilage injuries, and in five patients, acetabular cartilage injuries were present. A torn of the ligamentum teres was noted in all patients. All injuries were addressed arthroscopically. 11 In the present case, with an Outerbridge III defect, presenting a fissuring at the level of the subchondral bone, we decided to remove the fragment without further surgical intervention such as chondroplasty or microfracture. Kocher et al described chondroplasty of the acetabulum or femoral head in 10 patients (< 18.9 years). In patients with Outerbridge IV degenerative changes, no improvement of the Harris Hip Score was reported after the procedure. 17 Philippon et al suggested microfracture for Outerbridge IV full-thickness damages in pediatric patients with femoroacetabular impingement. 18 They described microfracture for chondral defects in nine professional athletes after hip dislocation. 19 Similar to the mentioned studies, we were able to fully address the pathology in the 13-year-old boy using an arthroscopic approach without the necessity to convert to an open approach or further intervention. The hip remained stable after the procedure. Timing of the operative intervention is critical, in our patient we decided to schedule the arthroscopy for the next day after referral. The operation should be performed by a team, which is skilled in hip arthroscopy. In general we would not suggest a delay of more than 1 to 2 days after diagnosis of the nonconcentric reduction in patients with a small intra-articular bony fragment and labral tear. If gross dislocation persists, an immediate operation is indisputable.

Even though arthroscopic treatment appears to be less invasive, it leaves smaller scars compared with open approaches or surgical hip dislocations, and complications should not be underestimated or ignored. Complications may be due to direct manipulation, such as damage to the femoral or acetabular cartilage, nerve injuries, during insertion of the instruments, or secondary to traction to the hip during the procedure or fluid instillation. In the adult population, the incidence of heterotopic ossification after hip arthroscopy is reported between 1.0 and 6.3% 20 and the incidence of deep vein thrombosis has been reported to be 1.4%. 21 Kocher et al reported three cases of pudendal nerve paresthesias which resolved spontaneously and one broken guidewire, which was retrieved arthroscopically. 17

No standardized protocols regarding the postoperative management exist. Our patient was instructed to toe tip weight-bearing for 6 weeks with restriction to a maximum hip flexion of 60°. Wylie et al began physiotherapy at 2 weeks, his patients started with touchdown weight-bearing for 2 weeks with partial weight-bearing for another 2 weeks and discontinuation of the crutches at 4 weeks. Hyperextension and external rotation of the foot was permitted for 6 weeks. Full range of motion was allowed between 6 and 12 weeks. 11

## Conclusion

In our experience and review of the literature, labral tears and intra-articular bony fragments, which lead to nonconcentric reduction after a pediatric hip dislocation, can be safely and successfully addressed with a hip arthroscopy. Removal of bony fragments, reduction, sutures, or labral resections can be performed to gain a stable reduced hip. The procedure should be performed by experienced surgeons, who are capable of converting to an open approach, although the possibility is likely to be minimal.

## Notes

New Insights and the Importance for a Pediatric Surgeon From our experience and review of the literature, labral tears and intra-articular bony fragments, which lead to nonconcentric reduction after a pediatric hip dislocation, can be safely and successfully addressed with a hip arthroscopy.
